# The Health of the Workless

**Published:** 1924-08

**Authors:** 


### THE HEALTH OF THE WORKLESS.

Prolonged and widespread periods of unemployment have almost invariably been attended in the past by more or less serious deterioration of the health of those who have suffered from them. Insufficient and unsuitable food, mental anxiety and fears for the future inevitably take their toll alike upon physical strength and moral. It is easier to "run down" bodily or mentally than to pick up again, and the process of recuperation in such cases may be relatively prolonged, since there are not only the current demands upon the constitution, but serious arrears to be made up. Looking, then, at the unhappy experience of the past, it is good to find that, in one portion of the kingdom at least, the period of scarcity of work through which we are still passing has had no evil effects upon health. The annual report of the Scottish Board of Health, which has just been issued, shows that there has been no widespread physical distress in Scotland, that serious crime, so far from increasing, has diminished, and that there has been less excessive drinking. It is naturally not all *couleur de rose.* In the Clyde Valley there is a lowered standard of health among the class of mother that attends the Child Welfare Centres; and in Glasgow and Paisley children between one and five years of age are less well nourished and clothed than in previous years. The conclusion of the whole matter, however, is that, "thanks mainly to the comprehensive relief measures of the community, the three years of unemployment are not likely to leave any serious mark on the physique of the people."

There is no reason to suppose that the experience of England and Wales will be found to be less favourable than that of Scotland, which rests not only upon the ordinary statistics, but also upon the results of a special medical inquiry. In fact we know already that the death-rate in the United Kingdom, including the infant death-rate, was last year lower than ever. These are highly remarkable results to have been achieved amid circumstances that might have been expected to react in an exceptionally unsatisfactory way. The secret is to be found in the phrase we have quoted from the report, "the comprehensive relief measures of the community"—measures, it is fair to remember, to which the beneficiaries themselves contribute. We are often glibly assured that our industrial system is "rotten," that such benefits as it produces are absorbed by "capital," and that the "workers" are left in the lurch. Without embarking upon the tempestuous seas of politics, we are entitled to point out that this discredited system has splendidly safeguarded the health of the industrial population during a period of unemployment which for length and intensity has not been matched for a century, if ever. Unemployment insurance is a substantial burden upon industry and it is easily capable of abuse, but its results from the point of view of hygiene have fully justified the cost. To have kept the people fed and clothed and out of the hospital in conditions which have hitherto meant semi-starvation and acute mental distress is an achievement the favourable consequences of which will not be confined to the present generation.

